# Check-list of vascular plant communities on ironstone ranges of south-eastern Brazil: dataset for conservation

**DOI:** 10.3897/BDJ.6.e27032

**Published:** 2018-07-12

**Authors:** Flavio Fonseca do Carmo, Rubens Custódio da Mota, Luciana Hiromi Yoshino Kamino, Claudia Maria Jacobi

**Affiliations:** 1 Instituto Pristino, Belo Horizonte, Brazil; 2 Universidade Federal de Minas Gerais, Belo Horizonte, Brazil

**Keywords:** canga, collection gaps, endemic plants, metallophytes

## Abstract

**Background:**

Ironstone ranges are considered hotspots for higher plants α and β diversity. The lack of studies and the intense degradation of the ironstone ranges, due to mining, motivated us to compile, for the first time, a list of vascular plants collected on iron-rich derived substrates from ancient landscape of south-eastern Brazil. All existing records in the Brazilian Virtual Herbarium of Flora and Fungi for each of the 43 municipalities containing ironstone ranges were downloaded, resulting in 17,954 vouchers identified to the species level. We found 2,933 species belonging to 160 families and 818 genera.

**New information:**

For the first time, we identified 148 species mentioned in endangered flora official lists and 48 narrow endemic species. Collecting efforts must still be supported to properly sample the vegetation since, for 143 sites, less than 10 records/site were found. This dataset will assist with the indication of dozens of plant species whose threat criteria must be urgently assessed to subsidise public policies on the use and conservation of the Brazilian flora.

## Introduction

Ironstone ranges occur mainly in Brazil, Australia, South Africa and India and are predominantly comprised of rock blocks of banded iron formations - BIF - from the Archean and Paleoproterozoic ages (Souza and Carmo 2015, Hagemann et al. 2016). Flora knowledge of these ancient ecosystems is still incipient. Yet, some sites such as Quadrilátero Ferrífero and the Carajás Range (Brazil) and the Pilbara and Yilgarn Cratons (Australia) are considered hotspots (*sensu* Gibson et al. 2010) for higher plants α and β diversity.

In Brazil, covering the highest parts of iron mountains and, therefore, with even more restricted insular distribution, there are duricrusts known as *cangas*. These duricrusts originated from the intense weathering of BIF rock blocks and other lithotypes with high metal content. Some canga outcrops can be about 20-30 m thick, with records of origin that started at ca. 50 Ma, thus representing one of the oldest landscapes in Brazil (Dorr 1969, Monteiro et al. 2014, Salgado and Carmo 2015).

Plants in ironstone ranges are frequently subjected to abiotic stressors such as acid substrates, daily temperature variation, very shallow soils with reduced water availabilityand anomalous metal contents. In sites characterised by substrates (rock and soil) with natural anomalous metal contents, outstanding plant communities can be found, called metallophytes, which are able to tolerate metal toxicity (Baker et al. 2010). Due to these characteristics and their disjunct distribution, ironstone ranges are sources of taxonomic novelties and various endemic species (Jacobi et al. 2015, Gibson et al. 2015a).

In some Brazilian ironstone ranges, the mineralogical constitution of the rock outcrops can reach 90% of iron oxides – haematite Fe_2_O_3_ – and hydroxides – limonite FeO(OH).nH_2_O, in addition to high manganese and aluminium contents (Jacobi et al. 2015, Schaefer et al. 2015). Soils developed in these systems are practically deprived of exchangeable nutrients, the result of both intense lixiviation and the oxidic nature of the rocks, characterising an environment with extreme oligotrophy. When associated with forest formations, the soils have higher organic matter values, but the prevailing carbon is recalcitrant and, therefore, very resistant to microbial cycling (Schaefer et al. 2015). These conditions represent a restrictive environmental filter, determining the establishment of specialised plant communities (Gibson et al. 2015a, Carmo and Jacobi 2015, Silveira et al. 2015).

Carmo and Kamino (2015) researched reference databases (Scientific Electronic Library Online and *Portal de Periódicos da Capes*) with keywords associated with ironstone ranges and found that, out of 189 articles produced in 31 countries, published between 1962 and 2015, only 10% of the content were related to Botany works. On the other hand, 77% of the articles addressed Geology issues. The authors pointed out that this imbalance between contents could generate inadequate public policies regarding use of natural resources and biodiversity conservation programmes.

Intense loss and degradation of areas in ironstone ranges are associated with the exploitation of natural resources, since they are home to the largest global reserves of iron ore, in addition to manganese and bauxite (aluminium). This geo-economical peculiarity favours a concentration of megastructures for ore extraction, generating an enormous environmental liability (Butcher et al. 2008, Jacobi et al. 2011, Sonter et al. 2014). The consequences for plant populations, in addition to the massive habitat losses, have yet to be properly assessed.

In view of the lack of studies and rapid degradation of these unique ecosystems, the purpose of this study was to assemble, for the first time, a list of vascular plants collected on Fe-rich derived substrates from ancient ironstone ranges in south-eastern Brazil, updating their taxonomic nomenclature, indicating life forms and highlighting endemic taxa and threat categories. Additionally, we identified the main vegetation types and highlighted locations where inventory efforts must be encouraged.

## Materials and methods

### Geographical context

**Figure 1.**
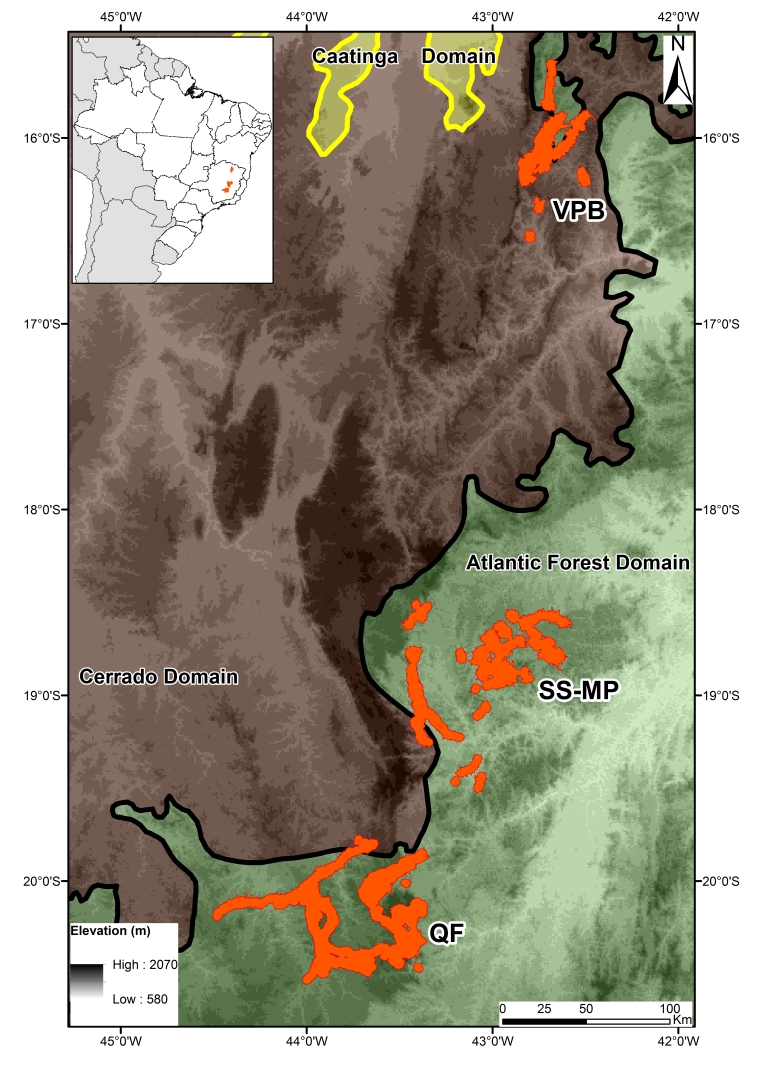
Ironstone ranges, south-eastern Brazil (orange polygons) as related to the Atlantic Rainforest and Cerrado phytogeographical domains. QF: Quadrilátero Ferrífero; SS-MP: Serra da Serpentina-Morro do Pilar; VPB: Peixe Bravo River Valley.

In south-eastern Brazil, ironstone ranges spread non-continuously over a 500 km-long north-south axis, in 43 municipalities distributed over three mineral provinces: Quadrilátero Ferrífero (QF) in the south; Serra da Serpentina-Morro do Pilar region (SS-MP) at the centre; and the Peixe Bravo River Valley region (VPB) in the north (Fig. 1).

In the QF and SS-MP ironstone ranges, there is a prevalence of the subtropical highland climate (Cwb) according to Köppen’s classification, characterised by dry winters and rainy summers (Davis et al. 2005, Souza and Carmo 2015). The vegetation matrix is comprised mainly of Brazilian Atlantic Rainforest, one of the most megadiverse and endangered rainforests in the world (Stehmann et al. 2009). In the VPB, dry weather with rainy summers (BSw) prevail. In these semi-arid conditions, the matrix is comprised predominantly by Cerrado, the Brazilian savanna, although there are still some spots of Brazilian Atlantic Forest. VPB is also in close contact with Caatinga, the largest seasonally dry tropical area of South America (Carmo et al. 2011). Comparisons between some of the physical and geographical variables can be seen in Table 1.

**Figure 2.**
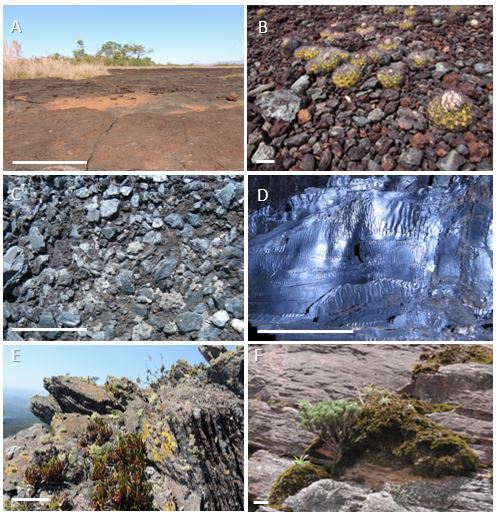
Heterogeneity of outcrops in ironstone ranges. **A-C**. Canga types (iron duricrusts); **D**. Specular hematite; **E-F**. Banded Iron Formations (BIF). The white bar represents 10 cm.

A peculiar feature of these ironstone ranges is the high physical heterogeneity, represented by regolithic materials and various types of outcrops (Fig. 2). The pronounced topographic heterogeneity in cangas may influence the proportion of functional plant groups, depending on the variation of substrate roughness (Carmo et al. 2015).

### Vascular plants dataset

**Figure 3.**
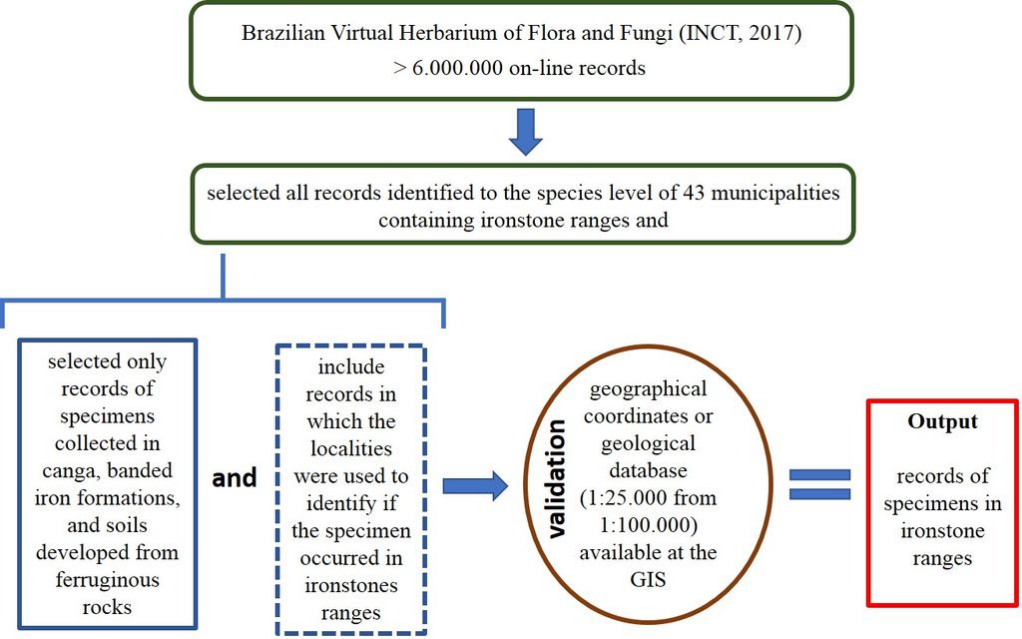
Construction and validation of vascular plants dataset in ironstone ranges of south-eastern Brazil.

All existing records in the Virtual Herbarium of Flora and Fungi (INCT 2017) for each of the 43 municipalities containing ironstone ranges were downloaded. We selected only the records of specimens collected in canga areas, banded iron formations and soils developed from ferruginous rocks. For the vouchers without information on substrate type, the collection localities were used to determine if the specimen occurred in ironstones. For this validation, the geological database (1:25,000 from 1:100,000) available at the webgis Atlas Digital Geossistemas Ferruginosos (Instituto Prístino 2018) was used, see Fig. 3.

The checklist of vascular plants in the ironstone ranges was composed by both native and naturalised plants and included only records identified to the species level. The current accepted nomenclature followed the List of Species of the Brazilian Flora (Flora do Brasil 2020 em construção 2017). Families and genera were listed in alphabetical order following Judd et al. (2008), APG (2016) and PPG I (Pteridophyte Phylogeny Group) I (2016). The herbaria were identified according to Thiers (2014), to the chronology of sampling and the main collectors.

### Vegetation types and life forms

We analysed the life form spectra based on five categories: trees, shrubs, subshrubs, lianas/vines and herbs, according to Carmo and Jacobi (2013) and the List of Species of the Brazilian Flora (Flora do Brasil 2020 em construção 2017).

Using voucher information, taxa were organised under three phytophysiognomies (*sensu* Pirani et al. 2003): forest, comprising semi-deciduous Seasonal Forest under the names Gallery Forest and *Capão*; savanna, including Cerrado and Carrasco; and grassland, covering Campo Limpo, Campo Sujo, vegetation associated with canga and BIF outcrops, seasonal water pools, ponds and bogs.

### Endemisms and species threatened with extinction

Complementary records and refined information on the sites of occurrence of the taxa, including cases of restricted endemicity, follow the list of studies presented in Suppl. material 1. Species with restricted geographic range and habitat specificity were deemed as narrow endemic species (*sensu* Rabinowitz 1981). For each taxon, we followed the threat category in the Official Lists of Endangered Species of the Minas Gerais and Brazilian Floras (Minas Gerais 1997, Brasil and Ministério do Meio Ambiente 2014).

## Analysis

### Vascular plants dataset

**Figure 4.**
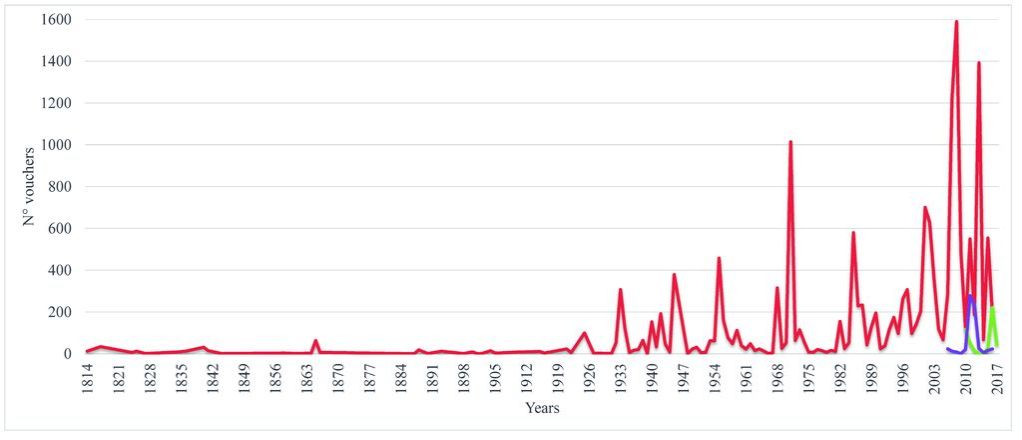
Timeline of the 17,954 records (vouchers) with identification to the specific level, for the ironstone ranges in south-eastern Brazil. Quadrilátero Ferrífero (red line); Serra da Serpentina-Morro do Pilar (purple line); Peixe Bravo River Valley (green line).

We found 17,954 records (vouchers) identified to the species level, originating predominantly from specimens collected in the QF ironstone ranges (93%), followed by samples from SS-MP (4%) and VPB (3%), see Fig. 4. The vouchers are deposited in 84 herbaria, of which 68 are Brazilian. The ones with the largest number of records were: BHCB (Herbarium of the Federal University of Minas Gerais, Brazil) with 9,222 (51%); NYBG_BR (The New York Botanical Garden, EUA) with 1,056 (5.9%) and OUPR ("Professor José Badini" Herbarium of the Ouro Preto Federal University, Brazil) with 1,026 (5.7%) records (Suppl. material 2).

The oldest records are from 1814, 1817 and 1824, collected by German naturalists Friedrich Sellow, Carl Friedrich Philipp von Martius and Ludwig Riedel. The years with the highest number of samples were (Fig. 4): 2008 (1,555), 2013 (1,414), 2007 (1,209) and 1971 (1,014). Vouchers were associated to 470 collectors, of which the main ones were, according to the number of samples: Carmo, F.F. (1,935), Mota, R.C. (709), Irwin, H.S. (685), Souza, F.S. (684), Roth, P.L. (623), Miranda, E. (594), Mello-Barreto (515), Viana, P.L (491), Rezende, S.G. (477) and Grandi, T.S.M. (435).

Of the 43 municipalities that contain ironstone ranges, only seven had more than 1,000 records each, all located in the QF. On the other hand, 11 municipalities had less than 10 records each and five of them had no vouchers. Of the 206 sites associated to Fe-enriched derived substrates, 10 alone concentrate 71% (12,515) of all vouchers (Table 2).

A total of 2,979 taxa were compiled, of which six were at the subspecies and 40 at the variety levels. Angiospermae accounted for 92% (2,737) of taxa, Monilophyta for 223 (7%), Lycophyta for 18 (0.6%) taxa and only one corresponds to Gimnospermae (Suppl. material 3). Several taxa were rare in herbaria, such as *Staurogyne warmingiana* (only 3 records); *Paepalanthus flaviceps* (4 records dated 1814); *Maytenus radlkoferiana* (7 records); *Mimosa pabstiana* (9 records); and *Stachytarpheta harleyi* (11 records). Another ten taxa were identified as naturalised species: *Amaranthus spinosus*, *Gymnanthemum amygdalinum*, *Tilesia baccata*, *Cerastium rivulare*, *Oeceoclades maculata*, *Plantago major*, *Andropogon gayanus*, *Hyparrhenia rufa*, *Panicum repens* and *Lantana camara*.

The 160 botanic families found account for 58% that occur in Brazil and 44 were represented by a single taxon. The 10 most representative families grouped 1,538 taxa (52% of the total): Asteraceae (387), Fabaceae (230), Poaceae (187), Orchidaceae (174), Melastomataceae (152), Rubiaceae (101), Cyperaceae and Myrtaceae (85 each), Apocynaceae (78) and Malpighiaceae (59).

The 818 genera account for 26% that occur in Brazil, of which 374 were represented by a single taxon. The 10 most representative grouped 358 taxa (12% of the total): *Mikania* (42), *Paspalum* (41), *Solanum* (40), *Paepalanthus* (39), *Baccharis* and *Miconia* (35 each), *Chamaecrista* (34), *Myrcia* (33), *Leandra* (30) and *Lessingianthus* (29).

### Life forms and vegetation types

**Figure 5.**
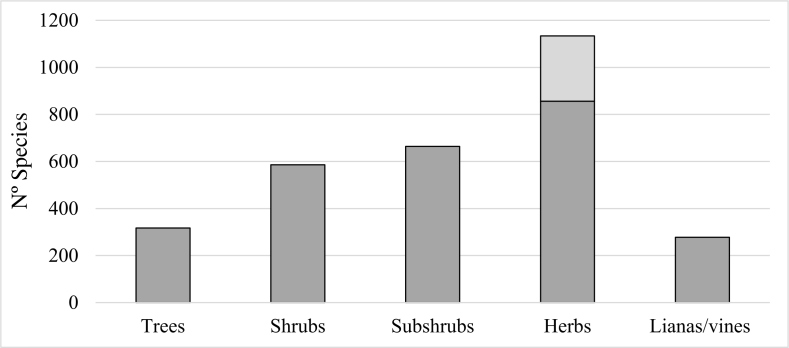
Life forms found in plant communities in the ironstone ranges of south-eastern Brazil. Light grey bar represents graminoid herbs.

The life forms (Fig. 5), with their respective numbers and taxa percentages, were: herbaceous (1,085 or 37.6%), of which 278 were graminoids; subshrubs (648 or 22.5%); shrubs (576 or 19.9%); trees (305 or 10.6 %); lianas/vines (278 or 9.4%).

Most taxa (1,436 or 48%) were concentrated on grassland physiognomies. The open vegetation, formed by plant communities associated with canga and BIF outcrops, was dominated by subshrubs and shrubs of Asteraceae, Euphorbiaceae, Fabaceae, Malphigiaceae, Melastomataceae, Velloziaceae and Verbenaceae. Common morphological features amongst several species were observed, such as microphily or coriaceous leaves and ericoid or imbricate phyllotaxis. Clones of desiccation-tolerant plants also occur, mainly of the genera *Trilepis* and *Vellozia*. In the substrate composed of regolithic materials (shallow, gravelly soils or small fragments of BIF and canga), the physiognomy was predominantly represented by graminoid herbs (such as Cyperaceae and Poaceae) and sclerophytic shrubs. In a lesser proportion, we also found communities associated with bogs and ponds, dominated by graminoid herbs of Cyperaceae, Eriocaulaceae, Poaceae and Xyridaceae.

We found 735 taxa (25%) in forest physiognomies. These included “vegetation islands” known as *capões*, developing on the organic material deposited in large fractures, depressions or caves in iron formations. Forest formations also occur along the drainage of the colluvial ramps and on the margins of waterways, places where the soil becomes less shallow and wetter. Arboreal and shrub species of Fabaceae, Lauraceae, Melastomataceae, Myrtaceae, Rubiaceae and Solanaceae are frequent. In the understorey, many Monilophyta, Lycophyta, Bromeliaceae, Orchidaceae and Piperaceae are found, as well as lianas/vines of Apocynaceae, Bignoniaceae and Dioscoreaceae.

**Figure 6.**
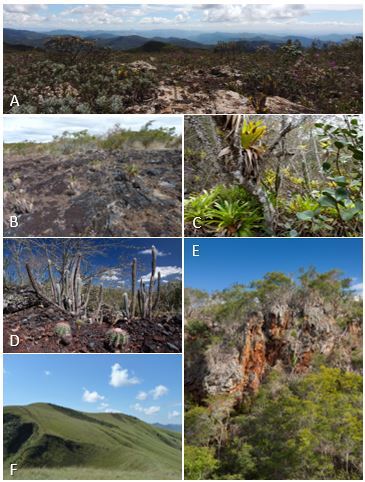
Vegetation types and phytophysiognomies in ironstone ranges. **A-B**. Plant communities in canga outcrops; **C**. Capão (Tree island); **D**. Carrasco vegetation; **E**. Atlantic Rainforest in contact with canga outcrop; **F**. Grasslands.

The recorded species number was low in savanna formations, as a result of fewer collections in these formations, particularly at VPB. In the Carrasco physiognomy, there is a prevalence of xeromorphic scrubs and deciduous trees of Euphorbiaceae, Fabaceae, Malvaceae and Salicaceae, in addition to columnar cacti (*Cereus* and *Pilosocereus*) that also occur in the Caatinga. The Cerrado occurs associated with ferruginous rock blocks/boulders found on slopes and at the top of some plateaus, with the shrub layer comprised of Asteraceae, Bignoniaceae, Calophyllaceae, Fabaceae, Lythraceae, Rubiaceae and Vochysiaceae. The herbaceous layer is also very developed and Apocynaceae, Cyperaceae and Poaceae species prevail. In addition, 645 taxa (21%) were collected in more than one physiognomy. Examples of some phytophysiognomies can be observed in Fig. 6.

### Endemisms and species threatened with extinction

The ironstone ranges hold exclusive records of 48 species (Table 2), mostly collected in the QF and all considered narrow endemic species. In addition to these, three species concentrate the records of ironstone substrates in the QF: *Calibrachoa elegans*, *Senecio linearilobus* and *Stachytarpheta confertifolia*, but each of these has only one disjunct record outside of ironstone ranges (Teles and Hattori 2012, Giacomin and Stehmann 2012, Salimena 2012). Taxa are contained in 20 families and 28 genera. The families with the largest number of endemic taxa were Bromeliaceae (13), Eriocaulaceae and Fabaceae (6 each) and Velloziaceae (5), totalling 61% of the endemic species. The most representative genera were *Dyckia* (10), *Paepalanthus* (6), *Barbacenia* (4) and *Mimosa* (3), comprising 47% of the exclusive taxa.

We found 148 species included in official lists of endangered flora (Suppl. material 3). Asteraceae (40), Lauraceae (11), Cactaceae (7), Fabaceae and Orchidaceae (6 each) were the families with the largest numbers of endangered species. Amongst genera, *Lychnophora* (8) and *Mikania* (6) stand out. The grassland physiognomies were home to 102 endangered species, most associated with canga and BIF outcrops; forest formations contained 27 species; the aquatic environments and savanna physiognomy registered only one species each. Seventeen endangered species occurred in more than one type of physiognomy/environment. Only 12 of the 48 narrow endemic species in ironstone ranges are mentioned in the official lists of endangered species. Probably seven species are extinct in the wild, caused by the loss of natural areas due to mining (Table 3).

## Discussion

The ironstone ranges in south-eastern Brazil stand out as areas of great value for the conservation of plant diversity. In a very restricted total area - less than 0.02% of the Brazilian territory - 2,933 species collected from Fe-rich substrates have been recorded so far. Although incomplete, this dataset already corresponds to 8% of all vascular plants occurring in the country, currently estimated at 34,475 (Flora do Brasil 2020 em construção 2017).

In the SS-MP and the VPB regions (see Fig. 1), collecting efforts must still be supported to properly sample the vegetation in these ironstone ranges since, for 143 sites (69% of the total), less than 10 records/site were found. Even for regions thoroughly sampled, notably the QF, intense efforts from plant taxonomists must be encouraged, since a relevant number of records still remains in herbaria without identification to the specific level (Jacobi and Carmo 2012). Amongst the material collected in some QF cangas (Carmo and Jacobi 2012), at least 10 taxa, deemed as new to science, were identified, but only one has been formally described (see Freitas Oliveira et al. 2014). As advocated by Mace (2004) and Callmander et al. (2005), promptly reducing collection gaps and increasing taxonomic efforts are needed to generate proper knowledge about the conservation status and levels of endemism of ironstone range species.

The phytogeographical context also favoured the high floristic diversity. The ironstone ranges in southeast Brazil receive influence from elements in the Atlantic Forest, Cerradoand Caatinga domains (see Fig. 1). Additionally, elements of Campos Rupestres (rupestrian grasslands) of the Espinhaço Range, one of the largest world centres of plant endemicity, have been found (Carmo and Jacobi 2013, Silveira et al. 2015).

A peculiar feature in iron-rich regions is the presence of plant communities characterised by species with remarkable ecological value due to their adaptation to metal substrates. In the ironstone ranges, some metalliferous ecotypes (*sensu* Whiting et al. 2004) have already been observed, such as *Eremanthus erythropappus* and *E. glomerulatus*, *Microlicia crenulata* and *Trembleya laniflora* (Teixeira and Lemos-Filho 1998). In addition, Felestrino et al. (2017)recently identified As-resistant bacteria associated with the roots and rhizosphere of a narrow endemic legume in the QF cangas, *Mimosa calodendron*. This finding creates opportunities to develop biotechnological research for soil rhizoremediation, notably in contaminated areas.

Within a global context, the α-diversity (2,933 spp.) and number of narrow endemic species (48 spp.) found in the ironstones of south-eastern Brazil have more representative values than those known for plant diversity hotspots in other metallicolous floras. In Australia, 44 species are known whose distribution is restricted to, or centred on, ironstones (BIF) located in Yilgarn and 20 endemic species to Pilbara Ranges. Together, these sites contain about 1,300 inventoried species (Gibson et al. 2015a, Gibson et al. 2015b). In the ironstone ranges from Serra de Carajás, Brazilian Amazon, Viana et al. (2016) estimate that at least 600 species will be recorded for the cangas, of which at least 40 are presumably endemic.

Conservation planning and the creation of public policies adequate for the rational use of natural resources in the ironstone ranges are increasingly more urgent. We identified 2,259 records (13% of the total), distributed amongst 27 sites, directly associated with mining complexes. Some type-sites have already been completely destroyed due to iron ore extraction, such as Cauê Peak (19°35’S, 43°13’W), while others are intensely degraded, such as Serra de Itatiaiuçu (20°7,5’S, 44°22’W), Serra da Serpentina/Sapo (18°54’S, 43°26’W), Serra do Curral (19°56’S, 43°53’W) and Serra do Itabirito (20°13’S, 43°51’W).

Attributes such as rarity and endemicity were observed, with several taxa under-represented in herbaria, some only by type-material collected over a century ago. With several species mentioned in the Catalog of Rare Brazilian Plants (Rapini et al. 2009, Jacobi et al. 2011), the ironstone ranges therefore represent sites of global interest, identified as Key Biodiversity Areas. Amongst the 48 narrow endemic species in the ironstone ranges, only 12 are mentioned in official endangered flora lists. This scenario does not reflect the current and intense extinction threat due to loss and degradation of natural areas caused by mining, resulting in probable extinction of several species. We stress the negligence of the State, both by failing to update the official lists - particularly the regional list (Minas Gerais 1997) - against that which is set forth by the Global Strategy for Plant Conservation (GSPC) programme and for reducing the already insufficient area of legally protected ironstone ranges (e.g. Minas Gerais 2017).

Therefore, the present database will assist with the indication of dozens of species whose threat criteria must be urgently assessed. These actions are essential because “Red Lists” are internationally recognised as a tool for defining the conservation status of species and populations. They are essential for subsidising environmental public policies and decision-making concerning the use, planning and conservation of natural resources (Gärdenfors et al. 2008, Callmander et al. 2005).

## Supplementary Material

Supplementary material 1 List of studies consulted to verify information on geographic distribution, localities and populations of the taxa associated with the ironstone ranges of south-eastern Brazil Data type: References
Brief description: The List contains references of the studies with complementary records and information on the sites of occurrence of the taxa, including cases of restricted endemicity in the ironstone ranges of south-eastern Brazil.
File: oo_216564.docx Carmo, F.F.; Mota, R.C.; Kamino, L.H.Y.; Jacobi, C.M.

Supplementary material 2 List of vouchers in the ironstone ranges in ironstone ranges, south-eastern Brazil Data type: Occurences
Brief description: List of 17.954 vouchers identified to the species levels, predominantly from specimens collected in the ironstone ranges of south-eastern Brazil: Quadrilátero Ferrífero (QF); Serra da Serpentina-Morro do Pilar region (SS-MP); and the Peixe Bravo River Valley region (VPB).
File: oo_205801.csv Carmo, F.F.; Mota, R.C.; Kamino, L.H.Y.; Jacobi, C.M.

Supplementary material 3 List of taxa in the ironstone ranges of south-eastern Brazil. Red List - BR (Brazil 2014) and MG (Minas Gerais 1997): VU: Vulnerable; EN: Endangered; CR: Critically Endangered; EX: Extinct. Data type: List of taxa
Brief description: List containing 2,979 taxa observed in the ironstone ranges of south-eastern Brazil, highlighting species included in official lists of endangered flora.
File: oo_205804.csv Carmo, F.F.; Mota, R.C.; Kamino, L.H.Y.; Jacobi, C.M.

## Figures and Tables

**Table 1.** Physical and geographical variables of ironstone ranges in south-eastern Brazil. QF: Quadrilátero Ferrífero; SS-MP: Serra da Serpentina-Morro do Pilar; VPB: Vale do Rio Peixe Bravo.

**Ironstone Ranges**	**Geological Group (age)**	**Maximum elevation**	**Yearly rainfall average**	**Original Area**	**Drainage Basin**
**QF**	Itabira/Cauê Formation (Paleoproterozoic)	1,800 m	1,400 mm	980 km^2^	São Francisco and Doce Rivers
**SS-MP**	Serra da Serpentina (Paleoproterozoic)	1,100 m	1,400 mm	250 km^2^	Doce River
**VPB**	Macaúbas, Nova Aurora Formation/Membro Riacho Poções (Neoproterozoic)	1,000 m	800 mm	350 km^2^	Jequitinhonha and Pardo Rivers

**Table 2.** Ten sites associated to ironstone ranges that concentrated the largest number of vouchers.

**Sites**	**Coordinates**	**Vouchers**
Serra da Piedade	19°49’S, 43°40’W	3,407
Serra do Curral	19°56’S, 43°53’W	1,761
Serra do Antônio Pereira	20°20’S, 43°29’W	1,495
Serra da Calçada	20°07’S, 43°59’W	1,403
Serra do Rola Moça	20°04’S, 44°02’W	1,062
Serra de Itabirito	20°13’S, 43°51’W	851
Serra da Moeda	20°18’S, 43°56’W	750
Serra do Gandarela	20°05’S, 43°41’W	742
Capão Xavier mining complex	20°03’S, 43°59’W	544
Capitão do Mato Mining complex	20°06’S, 43°57’W	500

**Table 3.** List of 48 narrow endemic species in ironstone ranges, south-eastern Brazil. Red List - BR (Brazil 2014) and MG (Minas Gerais 1997): VU: Vulnerable; EN: Endangered; CR: Critically Endangered; EX: Extinct. Life form: shr. (shrub), her. (herbaceous), sub. (subshrub), li. (lianas/vines). Region of occurrence: QF (Quadrilátero Ferrífero), SS-MP (Serra da Serpentina-Morro do Pilar). See references in Suppl. material 1.

**Family/Species**	**Red List**		**Life form**	**Region**	**Notes**
	**BR**	**MG**			
Acanthaceae *Staurogyne warmingiana* (Hiern) Leonard	EN	-	sub.	QF	
Apocynaceae *Minaria monocoronata* (Rapini) T.U.P. Konno & Rapini	CR	-	sub.	QF	Only known from two localities. Probably extinct in the wild due to the large number of iron-mining activities (Rapini 2012).
Asteraceae *Mikania badiniana* G.S.S. Almeida & Carv-Okano	-	-	sub.	QF	Only known from the type locality (Almeida and Carvalho-Okano 2010).
Bromeliaceae *Cryptanthus ferrarius* Leme & C.C.Paula	-	-	her.	QF	The main population will be extinct in a few years due to the large number of iron-mining activities (Leme and Paula 2009).
*Dyckia conceicionensis* O.B.C. Ribeiro & Leme	-	-	her.	SS/MP	Only known from the type locality (Ribeiro and Leme 2015).
*Dyckia consimilis* Mez	-	-	her.	QF	
*Dyckia densiflora* Schult. & Schult.f.	-	-	her.	QF	
*Dyckia elata* Mez	-	-	her.	QF	
*Dyckia ferrisincola* O.B.C. Ribeiro & Leme	-	-	her.	QF	Only known from the type locality (Ribeiro and Leme 2015).
*Dyckia incana* O.B.C. Ribeiro & Leme	-	-	her.	QF	Only known from the type locality (Ribeiro and Leme 2015).
*Dyckia inflexifolia* E.A.E. Guarçoni & M.A. Sartori	-	-	her.	SS/MP	Only known from the type locality (Guarçoni et al. 2012).
*Dyckia rariflora* Schult. & Schult.f.	EN	-	her.	QF	
*Dyckia schwackeana* Mez	-	-	her.	QF	
*Dyckia simulans* L.B.Sm.	-	-	her.	QF	
*Vriesea longistaminea* C.C.Paula & Leme	CR	-	her.	QF	Only known from the type locality (Leme and Paula 2004).
*Vriesea minarum* L.B.Sm.	EN	-	her.	QF	
Cactaceae *Arthrocereus glaziovii* (K.Schum.) N.P.Taylor & Zappi	EN	VU	sub.	QF	
Caryophyllaceae *Paronychia fasciculata* Chaudhri	-	-	her.	QF	
Convolvulaceae *Jacquemontia linarioides* Meisn.	-	-	li.	QF	
Eriocaulaceae *Paepalanthus amoenus* (Bong.) Körn.	-	-	her.	QF	
*Paepalanthus argillicola* Silveira	-	-	her.	QF	Only known from type material (Echternacht et al. 2012), described almost 100 years ago. Probably extinct in the wild.
*Paepalanthus batatalensis* Silveira	-	-	her.	QF	Only known from type material (Echternacht et al. 2012), described almost 100 years ago. Probably extinct in the wild.
*Paepalanthus gomesii* Silveira	-	-	her.	QF	Only known from type material (Giulietti et al. 2009). Probably extinct in the wild.
*Paepalanthus moedensis* Silveira	-	-	her.	QF	Only known from type material (Echternacht et al. 2012), described almost 100 years ago. Probably extinct in the wild.
*Paepalanthus pallidus* Silveira	-	-	her.	QF	Only known from material type (Echternacht et al. 2012), described almost 100 years ago. Probably extinct in the wild.
Euphorbiaceae *Croton serratoideus* Radcl.-Sm. & Govaerts	-	-	sub.	QF	
Fabaceae *Chamaecrista itabiritoana* (H.S.Irwin & Barneby) H.S.Irwin & Barneby	-	-	shr.	QF	
*Chamaecrista secunda* (Benth.) H.S.Irwin & Barneby	-	-	sub.	QF	
*Lupinus laevigatus* Benth.	EN	EN	sub.	QF	Currently, known from small populations in the Serra do Rola Moça.
*Mimosa calodendron* Mart. ex Benth.	-	-	shr.	QF	
*Mimosa multiplex* Benth.	-	-	shr.	QF	Only known from the two type localities (Dutra and Garcia 2014).
*Mimosa pogocephala* Benth.	-	-	shr.	QF	
Gesneriaceae *Sinningia rupicola* (Mart.) Wiehler	EN	VU	her.	QF	
Lauraceae *Cinnamomum quadrangulum* Kosterm.	VU	VU	shr.	QF	
Melastomataceae *Microlicia formosa* Cham.	-	-	sub.	QF	
*Microlicia cuspidifolia* Mart. ex Naudin	CR	-	sub.	QF	
*Pleroma ferricola* A.L.F.Oliveira, R.Romero & P.J.F.Guim.	-	-	sub.	QF	
*Trembleya rosmarinoides* DC.	-	-	shr.	QF	
Orchidaceae *Cattleya milleri* (Blumensch. ex Pabst) Van den Berg	CR	-	her.	QF	
*Gomesa gracilis* (Lindl.) M.W. Chase & N.H.Williams	-	-	her.	QF	
Poaceae *Paspalum brachytrichum* Hack.	-	CR	her.	QF	
Simaroubaceae *Simaba suaveolens* A.St.-Hil.	CR	EX	shr.	QF	Only known from type material described in 1823 (Pirani 2009). Probably extinct in the wild.
Velloziaceae *Barbacenia cyananthera* L.B.Sm. & Ayensu	-	-	her.	QF	
*Barbacenia itabirensis* Goethart & Henrard	-	-	her.	QF	
*Barbacenia rubra* L.B.Sm.	-	-	her.	QF	
*Barbacenia williamsii* L.B.Sm.	-	-	her.	QF	
*Vellozia sellowii* Seub.	-	-	her.	QF	
Xyridaceae *Xyris villosicarinata* Kral & Wand.	-	-	her.	QF	
