# Supplementary material for: Check-list of vascular plant communities on ironstone ranges of south-eastern Brazil: dataset for conservation
Source: Biodivers Data J. 2018 Jul 12;(6):e27032. doi: 10.3897/BDJ.6.e27032 (PMC6053471; doi:10.3897/BDJ.6.e27032)
Supplement: Supplementary material 1 — List of studies consulted to verify information on geographic distribution, localities and populations of the taxa associated with the ironstone ranges of south-eastern Brazil [file bdj-06-e27032-s001.docx]

Supplementary data 1

List of studies consulted to verify information on geographic distribution, localities and populations of the taxa associated with the ironstone ranges of south-eastern Brazil.

Almeida, G.S.S. & Carvalho-Okano, R.M. 2010. Three new species of Eupatorieae (Compositae) from Brazil. Kew Bulletin 65: 255-261.

Barneby, R.C. 1991. Sensitivae censitae: a description of the genus *Mimosa* Linnaeus (Mimosaceae) in the new world. Memoirs of the New York Botanical Garden 65:1-835.

Borsali, E.F. 2012. A Flora Vascular Endêmica do Quadrilátero Ferrífero, Minas Gerais, Brasil: Levantamento das Espécies e Padrões de Distribuição Geográfica. 194 f. Dissertação (Mestrado) - Universidade Federal de Minas Gerais, Belo Horizonte - MG.

Braz, D.M. 2005. Revisão Taxonômica de *Staurogyne* Wall. (Acanthaceae) nos Neotrópicos. 228 p. Tese (Doutorado em Ciências Biológicas) - Instituto de Biociências da Universidade Estadual Paulista “Júlio de Mesquita Filho”, Campus de Rio Claro, Rio Claro. Available in: <http://hdl.handle.net/11449/100646>.

Büneker, H.M., Soares, K.P., & De Assis, L.C. 2016. The *Dyckia sordida* complex (Bromeliaceae, Pitcairnioideae) and a new species from Minas Gerais, Brazil. Phytotaxa 244: 57-68.

Cordeiro, I. 2012. Euphorbiaceae. In: Jacobi, C.M. & Carmo, F.F. (orgs.). Diversidade Florística nas Cangas do Quadrilátero Ferrífero. Belo Horizonte: Ed. IDM. p. 122-123.

Dutra, V.F., & Garcia, F.C.P. 2014. *Mimosa* L.(Leguminosae-Mimosoideae) dos campos rupestres de Minas Gerais, Brasil. Iheringia. Série Botânica 69: 49-88.

Echternacht, L., Trovó, M. & Sano, P.T. 2012. Eriocaulaceae. In: Jacobi, C.M. & Carmo, F.F. (orgs.). Diversidade Florística nas Cangas do Quadrilátero Ferrífero. Belo Horizonte: Ed IDM. pp. 114-119.

Giacomin, L.L. & Stehmann, J.R. 2012. Solanaceae. In: Jacobi, C.M. & Carmo, F.F. (orgs.). Diversidade Florística nas Cangas do Quadrilátero Ferrífero. Belo Horizonte: Ed IDM. pp. 190-194.

Giulietti, A.M.; Andrade, M.J.G; Trovó, M., Sano, P.T. 2009. Eriocaulaceae. In. Giulietti, A.M.; Rapini, A.; Andrade, M.J.G; Queiroz, L.P. & Silva, J.M.C. (Org.). Plantas Raras do Brasil. Belo Horizonte, MG. Conservation International, pp. 166-180.

Guarçoni, E.A.E; Sartori, M.A. & de Paula, C.C. 2012. *Dyckia inflexifolia* (Bromeliaceae), a New Species from Brazil. Annales Botanici Fennici 49: 407-411.

Jacobi, C.M. & Carmo, F.F. 2012. Floristic diversity of the Quadrilátero Ferrífero Cangas. Belo Horizonte: Ed IDM. p.222.

Leme, E.M.C. & Paula, C.C. 2004. Two new species of Brazilian Bromeliaceae. Vidalia 2:21-29.

Leme, E.M.C. & Paula, C.C. 2009. *Cryptanthus ferrarius*, a new species from the iron-rich soils of Minas Gerais, Brazil, on the way to extinction. Journal of the Bromeliad Society 59:104-108.

Lorea-Hernández, F.G. 1996. A systematic revision of the Neotropical species of *Cinnamomum* Schaeffer (Lauraceae). Saint Louis: University of Missouri.

Mello-Silva, R. 2012. Velloziaceae. In: Jacobi, C.M. & Carmo, F.F. (orgs.). Diversidade Florística nas Cangas do Quadrilátero Ferrífero. Belo Horizonte: Ed IDM. pp. 200-203.

Monteiro, R. & Gibbs, P.E. 1986. A Taxonomic Revision of the Unifoliolate Species of *Lupinus* (Leguminosea) in Brazil. Notes RBG Edinb. 44: 71-104.

Mota, N.F.O. & Viana, P.L. 2012. Gesneriaceae. In: Jacobi, C.M. & Carmo, F.F. (orgs.). Diversidade Florística nas Cangas do Quadrilátero Ferrífero. Belo Horizonte: Ed IDM. pp. 129-131.

Mota, N.F.O. & Wanderley, M.G.L. 2012. Xyridaceae. In: Jacobi, C.M. & Carmo, F.F. (orgs.). Diversidade Florística nas Cangas do Quadrilátero Ferrífero. Belo Horizonte: Ed IDM. pp. 211-214.

Mota, R.C., Batista, J.A.N. & Borba, E.L. 2012. Orchidaceae. In: Jacobi, C.M. & Carmo, F.F. (orgs.). Diversidade Florística nas Cangas do Quadrilátero Ferrífero. Belo Horizonte: Ed IDM. pp. 158-162.

Oliveira, A.L.F., Romero, R. & Guimarães, P.J.F. 2014. A new Brazilian species and some synonyms in *Pleroma* (Melastomataceae). Brittonia 66: 353-357.

Pirani, J.R. 2009. Simaroubaceae. In. Giulietti, A.M.; Rapini, A.; Andrade, M.J.G; Queiroz, L.P. & Silva, J.M.C. (Org.). Plantas Raras do Brasil. Belo Horizonte, MG. Conservation International, p. 374.

Queiroz, L.P. & Cardoso, D. 2012. Fabaceae. In: Jacobi, C.M. & Carmo, F.F. (orgs.). Diversidade Florística nas Cangas do Quadrilátero Ferrífero. Belo Horizonte: Ed. IDM. pp. 124-127.

Rapini, A., Silva, R.F.S., Sampaio, L.N.P. 2009. Apocynaceae. In: Giulietti, A.M.; Rapini, A.; Andrade, M.J.G; Queiroz, L.P. & Silva, J.M.C. (Org.). 2009. Plantas Raras do Brasil. Belo Horizonte, MG. Conservation International, pp. 54-64.

Rapini, A. 2012. Apocynaceae. In: Jacobi, C.M. & Carmo, F.F. (orgs.). Diversidade Florística nas Cangas do Quadrilátero Ferrífero. Belo Horizonte: Ed IDM. pp. 72-75.

Ribeiro, O.B.C. & Leme, E.M.C. 2015. Three new species of *Dyckia* from iron rich outcrops of the Espinhaço Range, Minas Gerais, Brazil. Journal of the Bromeliad Society 65: 14-27.

Salimena, F.R.G. 2012. Verbenaceae. In: Jacobi, C.M. & Carmo, F.F. (orgs.). Diversidade Florística nas Cangas do Quadrilátero Ferrífero. Belo Horizonte: Ed. IDM. pp. 204-206.

Simão-Bianchini, R. 2012. Convolvulaceae. In: Jacobi, C.M. & Carmo, F.F. (orgs.). Diversidade Florística nas Cangas do Quadrilátero Ferrífero. Belo Horizonte: Ed. IDM. pp. 103-105.

Smith, L.B. & Ayensu, E.S. 1976. A revision of American Velloziaceae. Smithsonian Contributions to Botany 30: 1-172.

Teres, A.M. 2008. Contribuição ao estudo taxonômico da Tribo Astereae no Brasil e Senecioneae (Asteraceae) no Estado de Minas Gerais. Tese. ICB/UFMG. 260 pp.

Versieux, L.M. & Wendt, T. 2006. Checklist of Bromeliaceae of Minas Gerais, Brazil, with notes on taxonomy and endemism. Selbyana 27: 107-146.

Versieux, L.M. 2012. Bromeliceae. In: Jacobi, C.M. & Carmo, F.F. (orgs.). Diversidade Florística nas Cangas do Quadrilátero Ferrífero. Belo Horizonte: Ed. IDM. pp. 93-97.

Viana, P.L. 2012. Poaceae. In: Jacobi, C.M. & Carmo, F.F. (orgs.). Diversidade Florística nas Cangas do Quadrilátero Ferrífero. Belo Horizonte: Ed. IDM. pp. 171-178.

Zappi, D.C. & Taylor, N.P. 2012. Cactaceae. In: Jacobi, C.M. & Carmo, F.F. (orgs.). Diversidade Florística nas Cangas do Quadrilátero Ferrífero. Belo Horizonte: Ed. IDM. pp. 98-100.
